# Microglial response to experimental periodontitis in a murine model of Alzheimer’s disease

**DOI:** 10.1038/s41598-020-75517-4

**Published:** 2020-10-29

**Authors:** Alpdogan Kantarci, Christina M. Tognoni, Wael Yaghmoor, Amin Marghalani, Danielle Stephens, Jae-Yong Ahn, Isabel Carreras, Alpaslan Dedeoglu

**Affiliations:** 1 Forsyth Institute, 245 First Street, Cambridge, MA 02142 USA; 2 Department of Veterans Affairs, VA Boston Healthcare System, Research and Development Service, Building 1A-(151), 150 S. Huntington Avenue, Boston, MA 02130 USA; 3 Department of Neurology, Boston University School of Medicine, Boston, MA 02118 USA; 4 Department of Radiology, Massachusetts General Hospital and Harvard Medical School, Boston, MA 02114 USA; 5 Department of Biochemistry, Boston University School of Medicine, Boston, MA 02118 USA

**Keywords:** Periodontitis, Alzheimer's disease, Inflammation

## Abstract

Periodontal disease (PD) has been suggested to be a risk factor for Alzheimer’s disease (AD). We tested the impact of ligature-induced PD on 5xFAD mice and WT littermates. At baseline, 5xFAD mice presented significant alveolar bone loss compared to WT mice. After the induction of PD, both WT and 5xFAD mice experienced alveolar bone loss. PD increased the level of Iba1-immunostained microglia in WT mice. In 5xFAD mice, PD increased the level of insoluble Aβ42. The increased level in Iba1 immunostaining that parallels the accumulation of Aβ in 5xFAD mice was not affected by PD except for a decrease in the dentate gyrus. Analysis of double-label fluorescent images showed a decline in Iba1 in the proximity of Aβ plaques in 5xFAD mice with PD compared to those without PD suggesting a PD-induced decrease in plaque-associated microglia (PAM). PD reduced IL-6, MCP-1, GM-CSF, and IFN-γ in brains of WT mice and reduced IL-10 in 5xFAD mice. The data demonstrated that PD increases neuroinflammation in WT mice and disrupts the neuroinflammatory response in 5xFAD mice and suggest that microglia is central to the association between PD and AD.

## Introduction

Alzheimer's disease (AD) is an age-associated neurodegenerative disorder and the most common type of dementia. AD is clinically characterized by a progressive decline in memory, language, and learning capacity, ultimately ending in death^1^. The pathological hallmarks of AD are the formation of extracellular plaques composed primarily of amyloid-beta peptide (Aβ) and intraneuronal neurofibrillary tangles (NFT) of hyperphosphorylated tau protein, which lead to the loss of neuronal synapses and neuronal degeneration^1,2^. AD pathology is associated with a sustained and unresolved neuroinflammatory response that is characterized by the chronic activation of microglia. In a mouse model of AD, we have recently demonstrated that the inflammatory process is impaired and can be restored by treatment with the agonists of resolution of inflammation^3^. Under homeostatic conditions, microglia cells are highly branched, surveying the environment for the detection of potential pathological changes. When this occurs, microglia undergo changes in morphology, surface phenotype, and secretory profile in a process referred to as activation^4^. Activated microglia are critical for the phagocytosis and clearance of foreign particles and cellular debris as well as for regulating the immune systems during the inflammatory response^5–7^. Microglia cells become activated in response to Aβ accumulation, cluster around Aβ plaques, and contribute to Aβ clearance^8^. However, the ability of microglia to clear Aβ may wane with age^9,10^. The chronic inflammatory response of microglia to the persistent accumulation of Aβ contributes to the disease progression by releasing neurotoxic cytokines and reactive oxygen species, which initiate a pro-inflammatory signaling cascade that exacerbates the deleterious effects caused by Aβ and tau^8,11^. Recent evidence suggests that plaque-associated microglia (PAMs) form a protective physical barrier around amyloid deposits, compacting amyloid fibrils into a potentially less toxic form, preventing the addition of new Aβ onto existing plaques, and protecting nearby neurons from Aβ toxicity^12^. Thus, both deleterious and protective effects of AD-induced activated microglia have been described with mounting evidence of the important role that microglia have in AD^13,14^.

Systemic inflammation is capable of inducing neuroinflammation^15,16^. Chronic inflammatory diseases have been significantly correlated with AD pathology^17–19^. Thus, the relationship between dissemination of inflammation from other pathologies in distant organs and AD progression is highly plausible. Chronic infections, such as periodontal disease (PD), which increase the overall “set point” of systemic inflammation, could modify the neuroinflammatory process^20–24^. PD is a chronic and multifactorial inflammatory process caused by microorganisms and characterized by progressive destruction of the tooth-supporting apparatus, leading to tooth loss^25,26^. Breakdown of periodontal tissues involves a complex interplay between the pathogenic bacteria, the microbial biofilm, and the host's immune responses^27^. During the establishment of PD, Gram-negative microorganisms increase up to 80%, colonize the gingival sulcus, form subgingival biofilm, and lead to the formation of periodontal pockets. In addition to aging and immunosenescence, chronic inflammation elicited by microbial infectious agents and their toxic products may also lead to aberrant microglia cell function^13,14^. The cell wall components and various toxic products of periodontal pathogens can trigger the host response and induce destruction of periodontal tissues^28^. Prevalence of PD is globally high making PD as one of the most common diseases^29^. PD is highly associated with aging. Almost 47% of adults aged 30 years or older in the United States have PD^30^. Chronic and systemically disseminating inflammation induced by PD is a risk factor for several conditions including stroke, cardiovascular diseases, diabetic complications, rheumatoid arthritis, and preterm birth^31^. A bi-directional link between AD and PD also has been suggested by cross-sectional human studies^21^. Tooth loss, which is a net result of progressive PD, was linked to cognitive decline and AD in the elderly^32–36^. Furthermore, a study reported the association of higher brain amyloid load and PD in a cognitively normal elderly cohort^37^. These studies demonstrate that PD results not only in the loss of bone support around the teeth but also in systemic inflammation; however, it is not known whether PD leads to changes in neuroinflammation nor the mechanism of PD-induced AD-associated pathologies.

In the present study, we tested the hypothesis that experimentally-induced PD in a mouse model of AD will impact the inflammatory process in the brain and microglia function. In contrast to other animal studies of PD that have used oral gavage or injections of human periodontal bacteria (e.g., *Porphyromonas gingivalis*), here we used a model of PD in which silk ligatures were placed on the maxillary second molars, resulting in the colonization of mouse oral bacteria and the development of PD rather than an introduction of a human pathogen. We thus examined differences in how ligature-induced PD results in alveolar bone loss and alters neuroinflammatory responses between AD-modeled mice and wild-type (WT) controls.

## Results

### Alveolar bone loss is higher in 5xFAD than in WT mice at baseline, and experimental PD increases bone loss in WT and 5xFAD mice

**Figure 1:**
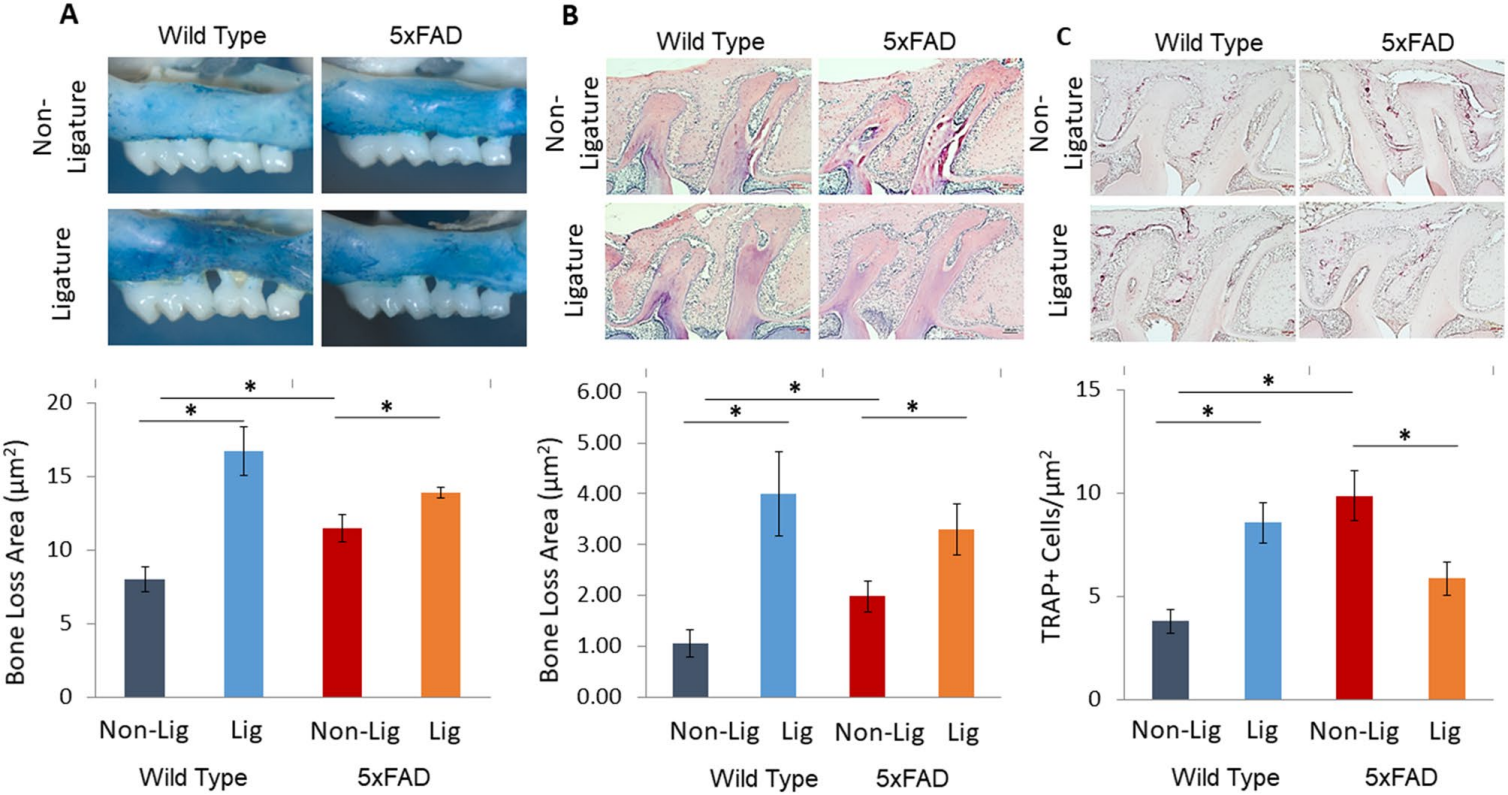
Experimental periodontitis and periodontal changes in a murine model of Alzheimer’s disease. (**A**) 5xFAD mice at baseline (not exposed to ligatures; Non-Lig) had higher level of bone loss than WT littermates. After induction of experimental periodontitis by the placement of ligatures (Lig), 5xFAD and WT mice both showed significant bone loss while no significant differences were detected between the level of bone loss in ligature-treated 5xFAD mice and ligature-treated WT mice. (**B**) Macroscopic findings were confirmed with histological measurement of alveolar bone levels in furcation region of the maxillary molar. (**C**) TRAP stained osteoclastic cells were significantly increased in 5xFAD mice compared to the WT mice at baseline. (*p < 0.05).

Silk ligatures were placed for four weeks on the maxillary second molars of 8-month-old 5xFAD and WT mice to induce PD. Since bone loss in the alveolus surrounding and supporting the teeth in the jaw is the hallmark of PD, we first measured the alveolar bone levels at baseline and after 4 weeks of placing the ligatures. As shown in Fig. 1A, the macroscopic examination of the maxilla of 5xFAD and WT littermates indicated that 5xFAD mice at baseline (not exposed to ligatures) had significantly higher levels of bone loss than WT littermates. After the induction of experimental PD, both 5xFAD and WT mice showed significantly increased bone loss, such that the difference between the bone loss area in 5xFAD and WT mice was no longer significant. Macroscopic findings were confirmed with histological measurements of alveolar bone levels in the furcation region of the maxillary molars in hematoxylin and eosin (H&E) stained sections (Fig. 1B).

To determine if the detected bone loss was associated with increased osteoclastic activity, maxillary sections were stained with tartrate resistant acid phosphatase (TRAP) (Fig. 1C). In parallel with the clinical and histological observations, TRAP+ osteoclastic cells at baseline were significantly increased in 5xFAD mice compared to WT mice, suggesting that the alveolar bone loss of 5xFAD mice was associated with increased osteoclastic activity. After ligature-induced PD was established, TRAP+ osteoclastic cell counts were significantly increased in WT mice while they were significantly decreased in 5xFAD mice.

### Experimental PD increases insoluble Aβ42 in 5xFAD mice

**Figure 2:**
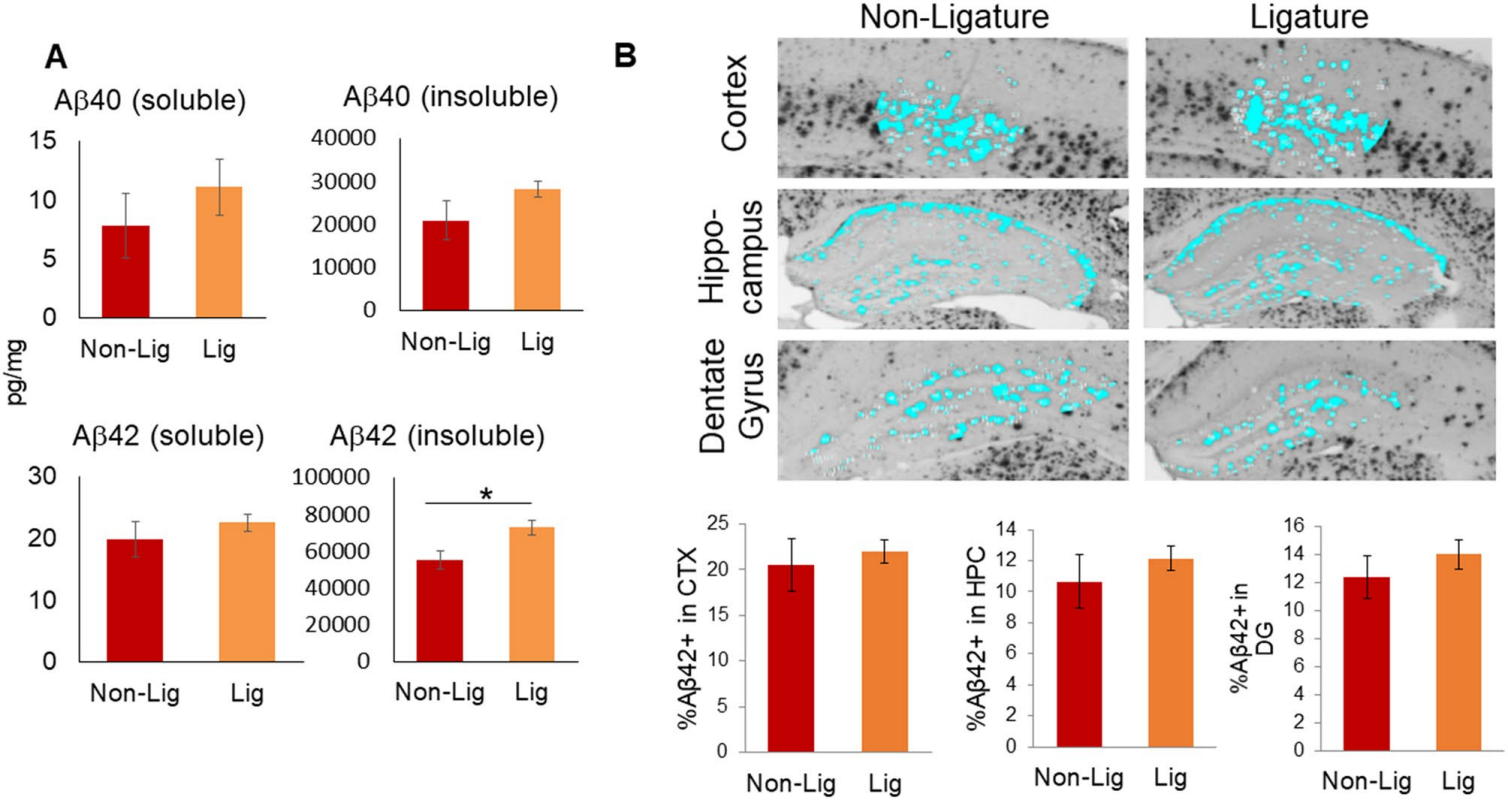
Amyloid beta (Aβ) accumulation and topographical distribution in the cortex, hippocampal formation, and dentate gyrus. (**A**) Soluble and insoluble forms of Aβ in brain extracts from 5xFAD mice with (Lig) or without (Non-Lig) experimental PD. *p < 0.05 compared to non-PD animals. (**B**) Photomicrographs of Aβ42-immunostained sections containing cortical layers 4–5 (CTX), the hippocampus (HPC), and the dentate gyrus (DG) subregion of the hippocampus, were taken with a 4 × objective from Non-Lig and Lig 5xFAD mouse brain sections. A constant threshold was applied to each image. Analysis of all thresholded Aβ42 particles was performed to obtain the percent area of Aβ42 staining.

To test if inducing experimental PD in 5xFAD mice leads to increased Aβ pathology, we measured the levels of Aβ40 and Aβ42 in the TBS-soluble and -insoluble protein fractions of the prefrontal cortex by ELISA and also quantified the plaque burden in brain sections immunostained with antibodies to Aβ42. The mean level of insoluble Aβ42, but not Aβ40, was significantly increased in ligature-treated 5xFAD mice compared to untreated 5xFAD mice (Fig. 2A). Differences of plaque burden measured in cortex, hippocampus, and dentate gyrus (DG) in ligature-treated and untreated 5xFAD mice were not significant (Fig. 2B).

### Experimental PD differentially affects the activation state of microglia in WT and 5xFAD mice

**Figure 3:**
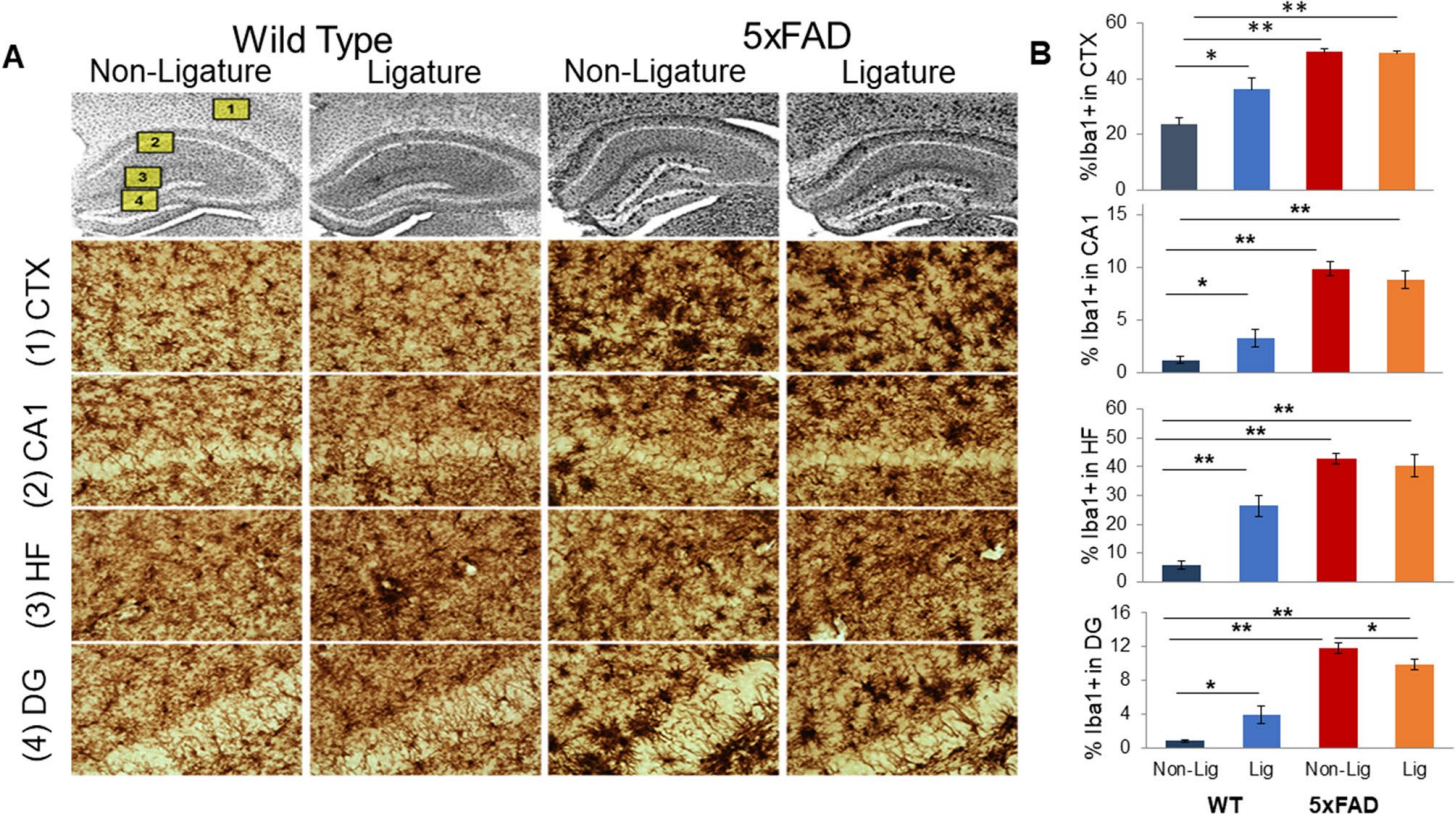
Impact of ligature-induced PD on microglia. (**A**) Photomicrographs of Iba1-immunostained sections from representative mouse brain sections at ×40 magnification (top row). Insets on the first image indicate the magnified (×400) regions-of-interest (ROIs) displayed below as (1) cortical layers 4–5 (CTX), (2) hippocampal CA1, (3) hippocampal fissure (HF), and (4) hippocampal dentate gyrus (DG). (**B**) Average microglia densitometry was analyzed for each mouse group within the CTX, CA1, HF, and DG ROIs, respectively. Using photomicrographs magnified at ×100, ROIs were outlined, a constant threshold was applied to each image, and analysis of all Iba1+ particles above the threshold was performed to obtain the percent area of Iba1+ staining (microglia densitometry). *p < 0.05, **p < 0.01.

We measured the impact of experimental PD on microglia activation in WT and 5xFAD mice by densitometric analysis of Iba1-immunostained brain sections. In WT mice, experimental PD led to significantly increased Iba1 levels in cortical layers 4–5 (CTX) and throughout the hippocampus, including the CA1, hippocampal fissure (HF), and DG (Fig. 3). As expected, 5xFAD mice had high levels of Iba1 immunostaining in cortex and hippocampus. Experimental PD in 5xFAD mice, however, did not lead to a further increase in Iba1 immunostaining and, in fact, resulted in a significant decrease in the DG compared to non-ligature 5xFAD mice.

**Figure 4:**
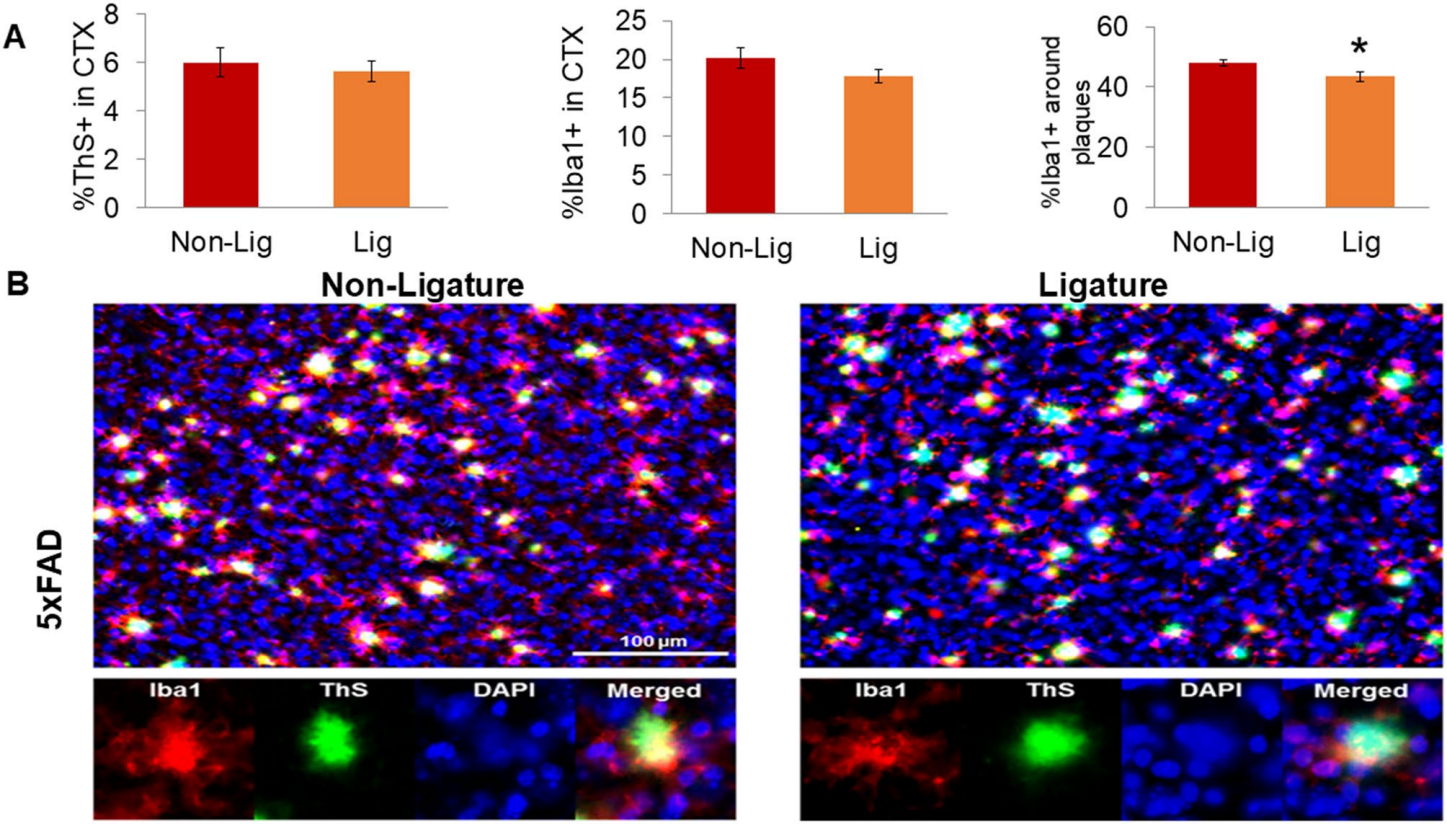
Impact of ligature-induced PD on plaque-associated microglia (PAMs) in 5xFAD mice. Fluorescent photomicrographs containing Iba1+ microglia (red), thioflavin S (ThS) dense-core plaques (green), and DAPI cell nuclei (blue) were taken of cortical layers 4–5 (CTX) from Non-Lig and Lig 5xFAD mice (×20 objective, 2 images/section, 3 sections/animal). Each fluorescent channel was automatically thresholded; an ROI around each ThS+ plaque (including a 6.5 μm buffer-zone) was generated; and channels were analyzed within and without the ROI. (**A**) Dense-core plaque densitometry (%ThS+), microglia densitometry (%Iba1+), and the percentage of PAMs (%Iba1+ staining within the immediate proximity of ThS+ plaques), *p < 0.05. (**B**) Representative fluorescent photomicrographs of Non-Lig (left panels) and Lig (right panels) 5xFAD mouse cortex. Scale bar = 100 μm. Bottom row: Iba1, ThS, DAPI, and merged channels from a representative image of a PAM.

Since function of activated microglia in AD depends on their association to Aβ plaques^12^, we examined if experimental PD altered the density of PAMs in 5xFAD mice. We performed double fluorescent labeling of brain sections from 5xFAD mice with and without PD with Iba1 antibodies to visualize microglia and with thioflavin S (ThS) to stain amyloid fibrils (Fig. 4). Double-label fluorescent images of CTX were processed to determine the total Iba1+ area of immunofluorescence and to define PAMs, as the percent area of Iba1 immunostaining in the immediate proximity (6.5 µm perimeter) of each ThS+ accumulation. We found that the percentage of PAMs was significantly decreased in 5xFAD mice with PD compared to naïve 5xFAD mice (Fig. 4A), indicating that development of PD results in less clustering of microglia around amyloid plaques. Consistent with the data from single-label immunostaining of Aβ42 (Fig. 2) and Iba1 (Fig. 3), there were no significant effects of experimental PD on total ThS fluorescence nor total Iba1 immunofluorescence (Fig. 4B).

### Analysis of Brain Cytokines

**Figure 5:**
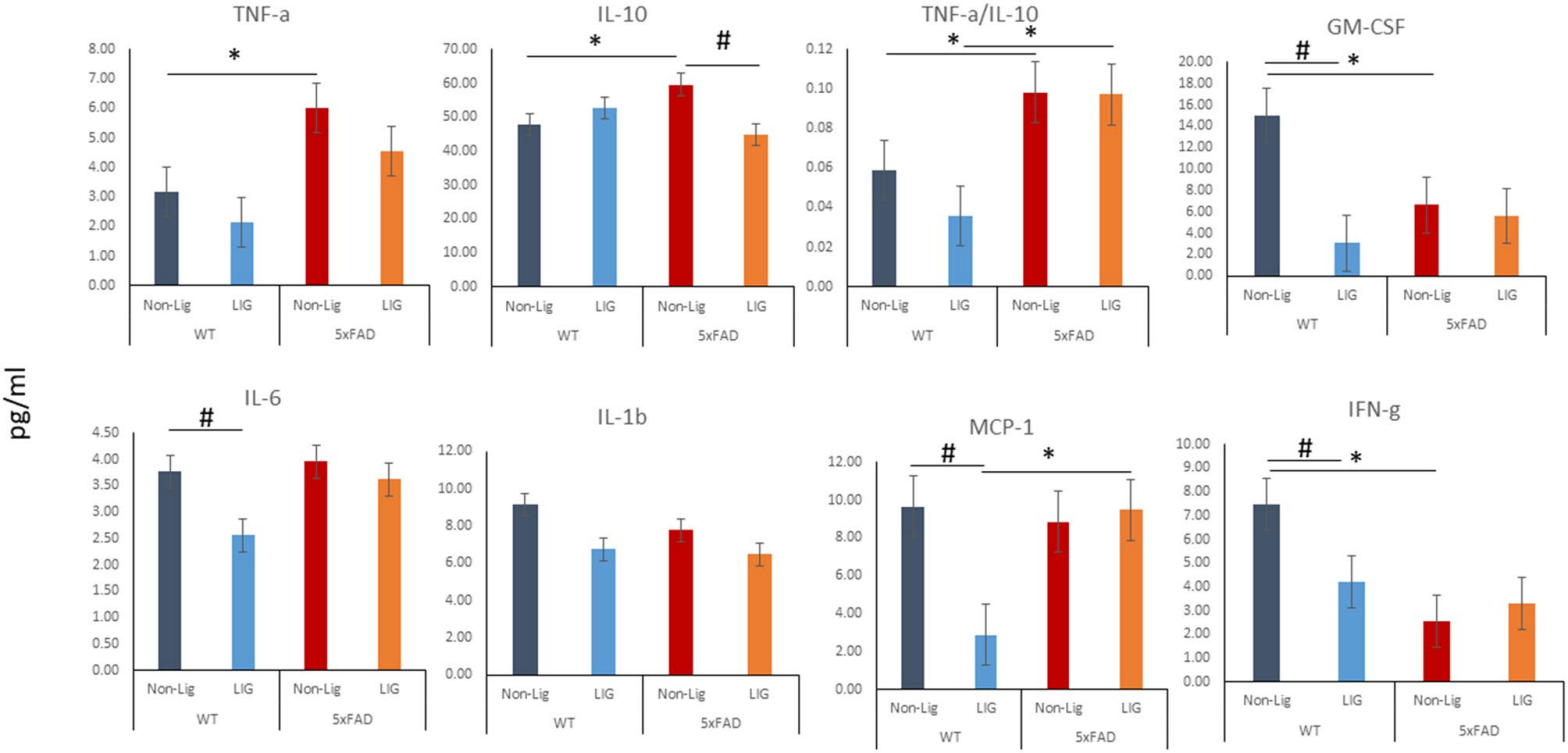
Cytokine profile of brain specimens in 5xFAD mice with or without ligature. Higher levels of TNF-α and IL-10, and lower levels of GM-CSF and IFN-γ in the 5xFAD mice brain samples compared to the WT controls were noted prior to the placement of ligatures. There was a significant reduction in IL-6, MCP-1, GM-CSF, and IFN-γ in brains of WT mice and IL-10 in 5xFAD mice after ligature placement. MCP-1 levels in ligated 5xFAD mice were significantly higher than in ligated WT mice. 5xFAD mice showed a higher and unresolved inflammation (TNF-α/IL-10 ratio) compared to WT controls (*p < 0.05, compared to WT animals; ^#^p < 0.05, compared to non-ligature group; ANOVA with posthoc analysis for multiple comparison).

We measured the concentration of cytokines in brain specimens of WT and 5xFAD mice with or without PD by multiplex immunoassay (Fig. 5). 5xFAD mice had significantly higher levels of TNF-α and IL-10 and lower levels of GM-CSF and IFN-γ in the brain samples compared to the WT controls in the absence of the placement of ligatures (p < 0.05). Ligature placement led to a significant reduction in IL-6, MCP-1, GM-CSF, and IFN-γ in brains of WT mice (p < 0.05) and in a significant reduction in IL-10 levels in 5xFAD mice (p < 0.05). MCP-1 levels in 5xFAD mice with PD were significantly higher than the WT mice (p < 0.05). When the ratio between TNF-α and IL-10 was compared as an index of inflammatory activation, 5xFAD mice showed a higher and unresolved inflammation in brain before and after the ligature placement compared to WT controls (p < 0.05).

## Discussion

In this study, we tested the hypothesis that experimentally-induced periodontitis using the ligature model will have an effect on the neuroinflammatory process in a mouse model of AD. We used a well-established mouse model of AD, which is characterized by rapid Aβ accumulation, and studied the impact of the PD, which represents a complex infecto-inflammatory disease of the oral cavity. The results demonstrated that the AD-like condition alone leads to periodontal bone loss and that experimental PD differentially affects brain inflammation in mice with AD-like pathology and WT mice. Furthermore, our data points to an aberrant activation of microglia and dysregulation of the neuroinflammatory process that could underlie the mechanism of a PD-AD link.

PD has already been associated with several severe diseases of other organs in the body including the cardiovascular diseases, diabetes, rheumatoid arthritis, and pre-term birth^38^. Recent data in humans has supported the premise that PD is associated with cognitive disorders and neuroinflammatory pathologies, including AD. In the context of pathological changes in the CNS associated with periodontal diseases in humans, the literature is growing, yet it is still not conclusive. A recent review summarized the current knowledge about the link between periodontal disease and cognitive disorders^39^. A recent systematic review presented evidence that stroke may be associated with poor oral health and periodontal disease^40^. Oral bacteria (e.g., *Porphyromonas gingivalis*) and their enzymes have been detected in the brain of AD patients and were associated with neuroinflammation and amyloid-beta deposition^28,41^. Collectively, these studies suggest that in humans, the oral microbiome has an impact on brain pathology, cognitive decline, amyloid and tau-associated neurodegeneration and neuroinflammation. There are several models of PD currently used in the field^42^. One of the models often used to study murine experimental PD is the oral gavage model in which a human periodontal bacterium such as *Porphyromonas gingivalis* is inoculated orally, after treatment with antibiotics to suppress the mouse oral microbiota. Using this model of PD, there are reports showing an increase in markers associated with AD pathology and cognitive deficits^43–46^. In the present study, we chose to use the ligature-induced model of PD. The advantage of the ligature-induced model over human-pathogen induced models is that a commensal mouse microbiome accumulates around silk ligatures placed around the mouse molars and colonize the periodontal space around the tooth. This leads to a switch in the pathogenicity of the periodontal microbiome as the species associated with periodontal disease dominate the microbial flora. Thus, as in humans, in the ligature-induced model, a transition from commensal to pathogenic microbiomes takes place in the periodontal tissue. The disease is mediated by an inflammatory process that in both humans and mice results in periodontal disease and tissue loss. As far as we know, this is the first study in a murine model that looks at the effect of ligature-induced PD in the brain.

There is also evidence that AD could be a risk for tooth loss and PD, suggesting a bi-directional effect between AD and PD^13,21,24,37,47–50^. Our data suggest a bi-directional link between the AD and PD because 8-month-old 5xFAD mice presented a significantly higher level of bone loss prior to the placement of ligatures and the induction of experimental PD. After the induction of experimental PD both 5xFAD and WT mice showed a significant increase in bone loss. The data showed a reduced osteoclastic activity, as demonstrated by the number of preosteoclasts and osteoclasts stained positive for TRAP, in 5xFAD mice with PD, while osteoclastic activity was increased in WT mice with PD. This finding suggested that the peak bone resorption as a result of the inflammatory process in the periodontal tissues may have taken place earlier in 5xFAD mice compared to the WT littermates and therefore time-dependent experiments would be needed to further understand the effects of PD. Another explanation of this observation may be a biological mechanism where PD may induce changes in the immune system responses and stimulate immunosenescence^51–53^, which could underlie the pathological process in AD. Evaluating the effects of ligature-induced PD in 5xFAD mice at earlier ages or in a slow-progressing AD models (e.g., PSAPP), may demonstrate the role of Aβ pathology in the link between AD and PD.

One of the key findings from this study is that experimental PD had an impact on microglia and the brain’s cytokine profile. The risk of developing PD increases with age; however, PD has a peak rate of occurrence in middle age that may result in priming microglia to a significantly activated state increasing the brain vulnerability to aging and to additional immune challenges or diseases. While microglia in WT mice was impacted by the experimental PD as shown by increased Iba1 expression, the level of inflammatory cytokines was also impacted, suggesting a defective immunological process due to PD^51–53^ that would lead to a disrupted inflammatory response. As PD can prime macrophages either through microbial factors (e.g., lipopolysaccharide)^54,55^ or by activation of other immune and non-immune cells^56,57^, microglia priming, activation, and polarization due to PD may be plausible^24^. The exaggerated response of primed microglia is known to contribute to the pathogenesis of AD^58^. In contrast to our previous study where we reported a significant increase in the level of some inflammatory cytokines (GM-CSF, IL-6, IFN-γ, and IL-1β) in 3-month-old 5xFAD mice^3^, in the current study using 8-month-old 5xFAD mice, these specific cytokines were not increased. Although we did not expect these results, a dynamic change of cytokines and pattern of different inflammatory markers throughout the disease course have been reported. For example, a very recent work^59^ suggested that microglia expressed multiple inflammatory profiles that change from 3 to 12 months of age and are associated with their protective response to accumulating Aβ plaques in the more slowly progressing APP/PS1 mouse model of AD. One study^60^ has raised the possibility that deposition of Aβ plaques initially buffer or diminish the brain immune response to the insult. Additional longitudinal studies will be necessary to evaluate this idea to understand this apparent discrepancy. Ligature-induced PD disrupts the brain’s cytokine profile especially in WT mice. As presented in Fig. 5, PD reduces the levels of IL-6, MCP-1, IFN-γ and GM-CSF in the brain of WT mice. Increasing number of studies support the concept that inflammatory dysregulation and cytokine imbalance is associated with neurodegenerative dementias^61^ and that rebalancing the activation of the innate immune system instead of suppressing it represent a new therapeutic approach. Therefore, the immune dysregulation that we detect after the development of PD may be of great significance and it will have to be further investigated.

In parallel with the increased insoluble Aβ42 levels, there was a significant decrease in plaque-associated microglia (PAMs) in 5xFAD animals with experimentally induced PD. This important finding suggests that the additional inflammatory challenge caused by PD results in a failure of microglia to function as a protective barriers around plaques, which opens hot spots around the plaques that result in the destabilization of plaques and the increased neurotoxicity^62^. Contrasting with the protective role of PAMs in Aβ phagocytosis and in preventing neuronal damage, dysfunctional PAMs can induce further neuroinflammation that disrupt non-PAMs microglia and other cells throughout the brain^63^. Thus, the effect of PD on PAMs can cause a significant modulation of neuroinflammation. Indeed, this observation was further supported by our findings that PD results in an aberrant profile of cytokines and chemokines, indicating a dysfunctional regulation of neuroinflammation in AD. While microglia cells ultimately become dysfunctional under AD conditions, the additional “hit” of PD may occupy some microglia to prevent PAMs from forming. Alternatively, PD might prime microglia such that they become more rapidly hyperactive and dysfunctional as AD pathology accumulates. Both of these possibilities, while further exploration is necessary, are highly plausible based on our findings that PD directly increases microglia activation, which was especially obvious in WT mice.

Our findings point to microglia as being a crucial link between the inflammation induced by PD and accelerated AD pathology. PD has been shown to result in a disruption in the inflammation and cytokine levels in brain. Since bacteria may infiltrate the central nervous system, which can directly activate microglia and indirectly via reactive astrocytes, PD may change the set-point of inflammatory modification of blood–brain-barrier and lead to a “leaky” barrier function. The mechanism of this effect is not known; however, the data clearly places PD at a risk level of that seen in other systemic inflammatory processes and suggests the possibility that oral microorganisms, which can cause systemic inflammation, should be regarded with the similar risk profile as the gut microbiome.

Collectively, we demonstrated that the alveolar bone loss was higher in 5xFAD than in WT mice at baseline, experimental PD increased bone loss in both 5xFAD and WT mice, experimental PD increased insoluble Aβ42 in 5xFAD mice and modified the microglia activation in both WT and 5xFAD mice, and experimental PD results in an aberrant inflammatory regulation in the brains of 5xFAD mice suggesting a distinct neuroinflammatory response to experimental PD in WT and 5xFAD mice and therefore a potential mechanism that can link PD and AD.

## Materials and methods

### Mice

Eight-month old male 5xFAD (n = 15) and non-transgenic male littermate (WT; n = 15) mice were used in the study. 5xFAD mice co-express human APP and PS1 with multiple FAD mutations [APP K670N/M671L (Swedish) + I716V (Florida) + V717I (London) and PS1 M146L + L286V] with specific neuronal expression driven by the Thy-1 promoter^64^. Eight mice in each group (WT or 5xFAD) were treated with ligatures to induce experimental PD while seven mice per group were left untreated.

### Experimental periodontitis in mice

Ligature-induced PD was established after placement of silk monofilament threads corresponding to 7.0-thickness (Perma-Hand silk, black braided Ethicon A182). The ligature was placed subgingivally on the maxillary right and left second molars with fine microsurgical instruments under ketamine (80 mg/kg, i.p.) and xylazine (16 mg/kg, i.p.) anesthesia. PD developed and reached chronicity in 4 weeks. The mice were weighed weekly during the study to assess health status and no differences in weight were detected between mice treated with ligatures and those left untreated. All animal experiments were carried in accordance with the NIH Guide for the Care and Use of Laboratory Animals and were approved by the local animal care committee at the Forsyth Institute.

### Assessment of periodontal disease and alveolar bone loss

After euthanasia, the maxillae were dissected free of muscle and soft tissue, keeping the attached gingiva intact. The maxilla was then split into two halves from the midline between the central incisors. The right half was taken for morphometric analysis and the left half was used for histological evaluation. For morphometric analysis, the right maxilla was defleshed and stained with methylene blue for the visual distinction between the tooth and bone based on the absorption of the blue dye by the two hard tissues with different calcification^65^. The bone level was quantified using Image Analysis (Image-Pro Plus 4.0; Media Cybernetics).

### Histomorphometric assessment of periodontal bone loss

The left half of the maxilla was decalcified and embedded in paraffin as described before^66^. Thin sections (5 μm) were cut and stained either with H&E for light microscopy and identification of the histological bone loss or with tartrate-resistant acid phosphatase (TRAP) to measure the osteoclastic activity as a reflection of number of preosteoclasts and osteoclasts stained positive for TRAP^67^. To further quantify the changes in the bone at the histological level, the mean value (± SD) of the linear distance and the area of bone loss was calculated as we have previously published^66^. A previously developed measurement technique was used to calculate the bone changes at three different sections of the root^66,68^. Linear distance was reported as the distance from the base of the epithelium to the alveolar crest border at the apical, middle, and the coronal third of the root and expressed as the difference between 5xFAD and WT animals. Area measurements were presented as the difference between the total area. Osteoclastogenesis was examined in TRAP-stained sections by counting the osteoclasts. 3,3–diaminobenzidinetetrahydrochloride (DAB)-stained slides were counterstained with hematoxylin. Images were acquired and analyzed using the Nikon Eclipse 80*i* microscope and Northern Eclipse Imaging Elements-D (NIS-D) software. Results were expressed as percent of total positive cells.

### Brain collection and processing

After euthanasia, brains were collected. The left hemisphere was post-fixed with 4% paraformaldehyde solution for 24 h and cryoprotected in a graded series of 10% and 20% glycerol/2% DMSO solution for the immunohistological analysis of microglia and Aβ plaques. The prefrontal cortex of the right hemisphere was dissected for the analysis of Aβ levels by ELISA and for the levels of inflammatory cytokines and chemokines.

### Analysis of Aβ deposits and microglia by immunohistochemistry

Cryoprotected hemibrains were serially cut at 50 μm on a freezing microtome and were immunostained with specific antibodies, as described^69^. Anti- Aβ1–42 (Invitrogen #700254, 1:1000) was used to define Aβ deposits and anti-Iba1 was used to stain microglia (Wako Chemicals #019-19741; 1:5000). In brief, free-floating sections were incubated overnight in primary antibody followed by PBS (Phosphate buffered saline) washes and incubation in peroxidase-conjugated secondary antibody followed by development using 3′-diaminobenzidine tetrahydrochloride (DAB) as a chromagen. Three serial sections per subject, 0.5 mm apart from each other were quantified in a blinded manner. Quantification of Aβ and Iba1 immunostaining was performed by densitometry using the threshold function on NIH Image J.

### Double-label fluorescent analysis of plaque-associated microglia (PAMs)

A series of brain sections underwent a 30 min antigen retrieval incubation at 60 °C in citrate buffer, followed by counterstaining of plaques, in which sections were incubated in 0.01% thioflavin S (ThS) in 40% ethanol for 45 min and then differentiated in 3 min with 40% ethanol. Sections then underwent immunostaining using the Iba1 primary antibody and a fluorescently-tagged secondary antibody (1:200 Texas Red goat anti-rabbit, Vector Labs) to label microglia and fluorescent staining of cell nuclei using DAPI. Multi-channel images of cortical layers 4–5 (2 images/section, 3 sections/animal) were taken using a 20 × objective on a fluorescent microscope (Nikon Eclipse 50i). Images were processed using NIH ImageJ by automatically thresholding the image of each channel and measuring the densitometry (% area) of Iba1 within the local proximity of ThS+ plaques (plaque-associated microglia, within a 6.4 µm enlarged radius of ThS staining) and within the space between plaques (non-plaque associated microglia).

### Analysis of Aβ levels by ELISA

Dissected pre-frontal cortex was homogenized with 10 volumes (w/v) of Tris-buffered saline (TBS) and centrifuged at 100,000*g* for 1 h at 4 °C. After saving the soluble supernatant fraction, the resulting pellet was re-suspended with 10 volumes of cold 5 M guanidine HCl buffer and saved as the insoluble fraction. Specific human Aβ40 and Aβ42 ELISA kits (Invitrogen, cat# KHB 3544 and KHB 3545) were used to analyze the soluble and insoluble tissue fractions according to the manufacturer’s specifications and as previously described^3^.

### Analysis of brain inflammatory cytokines and chemokines

Cortical brain homogenates were analyzed for a panel of inflammatory cytokines and chemokines (GM-CSF, IFN-γ, IL-1β, IL-6, IL-10, TNF-α, MCP-1) using the multiplex immunoassay, according to the manufacturer’s specifications (Millipore Sigma; MCYTOMAG-70 K) and as previously described^3^. The results were read on Bio-Plex 200 following manufacturers’ specifications and using Bio-Plex Manager software version 6.0 (Bio-Rad Laboratories, Hercules, CA).
